# Effect of Different Surface Treatment Protocols on Shear Bond Strength of Bulk-fill Resin Composite: an SEM Evaluation

**DOI:** 10.3290/j.ohpd.c_2841

**Published:** 2026-09-08

**Authors:** Ceyda Sarı, Cemile Kedici Alp, Sinem Akgül

**Affiliations:** a Ceyda Sarı Assistant Professor, Department of Restorative Dentistry, Faculty of Dentistry, İstanbul Medipol University, İstanbul, Turkey. Study concept and design, drafted the manuscript, conceived the ideas, performed statistical analysis, critically revised the manuscript, read and approved the final manuscript.; b Cemile Kedici Alp Associate Professor, Department of Restorative Dentistry, Faculty of Dentistry, Gazi University, Ankara, Turkey. Study concept and design, drafted the manuscript, conceived the ideas, collected and analyzed the data, critically revised the manuscript, read and approved the final manuscript.; c Sinem Akgül Associate Professor, Department of Restorative Dentistry, Faculty of Dentistry, Gazi University, Ankara, Turkey. Study concept and design, drafted the manuscript, collected the data, critically revised the manuscript, read and approved the final manuscript.

**Keywords:** CoJet, composite repair, SEM, surface conditioning.

## Abstract

**Purpose:**

To evaluate the repair bond strength of aged bulk-fill resin composites using various surface treatment protocols and analyze the composite–composite interface via scanning electron microscopy (SEM).

**Materials and Methods:**

One-hundred fifty disk-shaped specimens (4 mm height × 5 mm diameter) were prepared from three bulk-fill composites. After thermocycling (10,000 cycles, 5°C–55°C), specimens were assigned to five groups: (1) control (no treatment); (2) Universal adhesive (UA); (3) 37% phosphoric acid + UA; (4) silane + UA; and (5) CoJet + Silane + UA. Filtek Bulk-Fill Flowable was applied as the repair composite. After 24-h storage, shear bond strength (SBS) was measured, and one specimen per group was examined under SEM.

**Results:**

Two-way ANOVA showed statistically significant effects of both surface treatment and composite type on SBS (p < 0.05). CoJet + silane + UA produced the highest SBS values for all composites, whereas the untreated control produced the lowest values. No statistically significant difference was observed between UA and phosphoric acid + UA (p > 0.05), while silane + UA resulted in statistically significantly higher SBS values than both protocols. Material-dependent differences were observed in some treatment groups; however, no statistically significant differences among the three composites were detected following phosphoric acid + UA or CoJet + silane + UA treatment.

**Conclusion:**

Surface treatment statistically significantly improved the repair bond strength of aged bulk-fill composites. The combination of CoJet, silane, and universal adhesive provided the most effective bond enhancement.

Advancements in nanotechnology and adhesives have greatly improved the performance and clinical longevity of resin-based composites in restorative dentistry. These materials are widely used for esthetic anterior restorations as well as posterior load-bearing areas, driven by their enhanced mechanical properties and the growing demand for minimally invasive esthetic treatments across the dentition.^9,12^ Despite their popularity, resin composites still face intrinsic limitations, with polymerization shrinkage being a critical concern. This shrinkage can generate interfacial stress, marginal gaps, and microleakage, which may lead to secondary caries and restoration failure.^14^

To overcome the challenges of incremental layering and to simplify clinical procedures, bulk-fill resin composites were introduced. These composites allow placement in increments of up to 4–5 mm while maintaining adequate depth of cure and mechanical strength.^5,52^ Their improved efficacy is achieved by modifying filler loading, increasing translucency, and optimizing photoinitiator systems.^52^ Bulk-fill resin composites are generally divided into two types: low-viscosity (flowable) materials that require a capping layer, and high-viscosity (full-body) materials suitable for standalone use on occlusal surfaces.^25^

A subclass of bulk-fill resin composites, short fiber-reinforced materials (SFRC) susch as EverX Flow (GC; Tokyo, Japan) has been developed to mimic dentin’s biomechanical behavior. By incorporating randomly oriented glass fibers, these composites offer enhanced fracture toughness and better stress distribution, making them ideal for restoring structurally compromised or endodontically treated teeth.^9,25^

Filtek Bulk-Fill Flowable (3M Oral Care; St Paul, MN, USA) is another low-viscosity bulk-fill resin composite designed for deep cavity adaptation and simplified placement. Its resin matrix, composed primarily of bis-GMA, bis-EMA, and UDMA, is formulated to reduce viscosity and facilitate flow.^30^ Enhanced translucency and optimized photoinitiator systems allow polymerization in 4-mm increments, enabling rapid application in posterior restorations.^3^

Surefil SDR (Dentsply Caulk; Milford, DE, USA) is a flowable bulk-fill resin composite featuring a proprietary SDR (stress-decreasing resin) monomer designed to reduce polymerization stress during curing.^31^ The material exhibits favorable flow characteristics, high depth of cure, and improved adaptability to cavity walls; it is often used as a base material beneath conventional composites.^57^ Its stress-relieving resin matrix has been shown to facilitate more uniform polymerization, potentially enhancing bonding performance at the interface.^55^

Despite improvements in mechanical and optical properties, restorations inevitably fail over time due to fatigue, wear, marginal deterioration, and secondary caries. Traditionally, defective restorations have been fully replaced, which may unnecessarily remove healthy tooth structure, increase treatment time, and risk pulpal damage.^18^ In contrast, repair strategies provide a minimally invasive, cost-effective, and tissue-preserving alternative aligned with modern conservative dentistry principles.^38^

In cases where bulk-fill resin composites are used, particularly as base materials in extensive cavities or endodontically treated teeth, fractures may occur under functional load, exposing these materials to the oral environment. Although numerous studies have highlighted the potential of composite repair to extend the lifespan of restorations while preserving healthy tooth structure, the literature regarding the repairability of bulk-fill resin composites remains relatively limited.^1,11,15^

To improve adhesion at repair sites, various surface pretreatment strategies have been proposed, including mechanical methods like surface roughening with diamond burs or aluminum-oxide sandblasting, and chemical treatments such as phosphoric or hydrofluoric acid etching, silane application, and intermediate bonding agents.^15,18,35^ CoJet, a tribochemical silica coating system, produces a silica-coated, microretentive surface on resin-based substrates, thereby enhancing adhesion through both micromechanical interlocking and chemical bonding.^45^ Unlike conventional airborne-particle abrasion techniques that use pure alumina, the CoJet system utilizes 30-µm aluminum oxide particles that are surface-modified with approximately 30% silica.^6^ Universal adhesives containing functional monomers like 10-methacryloyloxydecyl dihydrogen phosphate (10-MDP) have shown enhanced micromechanical retention and chemical bonding to resin and inorganic fillers.^44^

Although surface treatment protocols for composite repair have been extensively described in the literature, evidence specifically addressing the repair performance of short fiber-reinforced flowable bulk-fill materials such as EverX Flowable remains limited. In particular, only a small number of studies have examined the influence of combined silane application as an additional chemical conditioning step within composite repair procedures, and most available reports focus primarily on conventional or high-viscosity bulk-fill resin composites rather than flowable SFRC systems.^2,16,40^ The present study aims to extend current knowledge by providing comparative bond strength outcomes for EverX Flowable under different repair protocols and by evaluating the potential contribution of additional silane use in aged composite substrates. Accordingly, the objectives of this study were to evaluate the repair bond strength of various aged bulk-fill resin composites under different surface treatment strategies. The null hypotheses were: (1) surface treatment has no significant effect on the repair bond strength, and (2) the type of bulk-fill resin composite does not influence the repair efficacy.

## MATERIALS AND METHODS

The compositions and the manufacturer details of the materials used in this study are shown in Table 1.

**Table 1:** Materials used in the study

Product Name	Composition	Lot number
Filtek Bulk-fill Flowable (3M Oral Care; St Paul, MN, USA)	Filler: zirconia or silica particles, ytterbium trifluoride particle Resin matrix: bis-GMA, bis-EMA, Procrylat resin, UDMA 64.5 wt%, 42.5 vol%	NC36727
EverX Flow (GC; Tokyo, Japan)	Filler: silicon dioxide, barium glass, glass fiber, polymethylmethacrylate and photo initiators Resin matrix: bis-GMA, TEG-DMA 70 wt%, 46 vol%	2312011
Surefil SDR (Dentsply Caulk; Milford, DE, USA)	Filler: barium, and strontium alumina-fluorosilicate glasses, photo initiators, coloring agents, strontium alumino-fluoro-silicate glass Resin matrix: EBPADMA, TEG-DMA, UDMA, dimethacrylate resin, difunctional diluents 68 wt%, 44 vol%	131024
Scotch Bond Universal (3M Oral Care)	10-MDP, HEMA, dimethacrylate resins, methacrylate-modified polyalkenoic acid copolymer, filler, ethanol, water, initiators, silane	40117A
Scotchbond Universal Etchant, (3M Oral Care)	37% phosphoric acid, syntheticamorphous silica (fumed), polyethylene glycol, aluminumoxide, water	582491
Ultradent silane coupling agent (Ultradent; South Jordan, UT, USA)	8% methacryloxy propyl trimethoxy silane <10%, isopropyl alcohol <95%	BRNZR
CoJet Sand (3M Oral Care)	Aluminium trioxide particles coated with silica, particles size: 30 µm	
10-MDP: 10-methacryloyloxydecyl dihydrogen phosphate; bis-GMA: bisphenol A glycidyl methacrylate; bis-EMA: ethoxylated bisphenol A glycidyl methacrylate; EBPADMA: ethoxylated bisphenol A dimethacrylate; HEMA: 2-hydroxyethyl methacrylate; UDMA: urethane dimethacrylate; TEG-DMA: triethylene glycol dimethacrylate.		

The required sample size was calculated using G*Power (version 3.1.9.6, Franz Faul, University of Düseldorf, Germany) with a significance level (α) of 0.05, a statistical power of 0.95, and an effect size of 0.86. A total of 150 disk-shaped specimens (height 4 mm x diameter 5 mm) were prepared with three different bulk-fill resin composite EverX Flow (GC) (n=50) and Surefil SDR (Dentsply) bulk-fill resin composite (n=50) and Filtek Bulk-fill flowable (3M Oral Caer) composite using polytetrafluoroethylene mold for the substrate group in A2 shade. To achieve smooth surfaces and prevent oxygen inhibition prior to polymerization, molds were placed between Mylar strips. The composites were placed in a single increment of 4 mm and polymerized for 20 s with a 1000 mW/cm^2^ LED curing light (D-Light Pro, GC). To ensure consistency in light curing, the same LED curing unit was used throughout the study. The light guide was positioned perpendicular and as close as possible to the specimen surface. Irradiance was maintained at 1000 mW/cm^2^ and verified using a radiometer (Bluephase Meter II, Ivoclar Vivadent; Schaan, Liechtenstein) after every five specimens.

Following polymerization and removal from the mold, the dimensions of each specimen were verified using a digital caliper (Insize 1112−150, Insize; Jiangsu, China) to ensure a diameter of 5 mm and a height of 4 mm. All specimens were also visually inspected for defects, including voids, cracks, edge defects, incomplete filling, or other visible irregularities. Specimens that did not meet the dimensional requirements or exhibited visible defects were excluded and replaced with newly prepared specimens. Following specimen preparation, the bottom surface of each specimen was marked with a scalpel to distinguish it from the active surface designated for subsequent surface treatment, repair, and bond strength testing after aging. The polymerized specimens were thermocycled (MTE 101, MOD Dental, Esetron Smart Robotechnologies: Ankara, Turkey) for 10,000 cycles at 5°C–55°C with a dwell time of 30 s and a transfer time of 10 s. After thermocycling, all specimen surfaces were subjected to a standardized roughening procedure using #320 silicon-carbide abrasive paper for 60 s at 200 rpm under water cooling. The specimens were embedded in polyvinyl chloride tubes using self-polymerizing polymethylmethacrylate (Paladent RR, Heraeus Kulzer; Hanau, Germany) and randomly divided into five groups (n=10) according to surface treatment protocols:

Group A: control; no treatment was applied to the repaired surface.
Group B: Universal adhesive; Scotchbond Universal (3M Oral Care) was actively applied to the composite surfaces using a microbrush with a rubbing motion for 20 s. The adhesive was then gently air thinned for 5 s using an oil-free air stream delivered through the three-in-one syringe of a dental unit and subsequently light cured for 10 s with an LED curing unit at an irradiance of 1000 mW/cm^2^.
Group C: 37% orthophosphoric acid + Universal adhesive; the surfaces were etched with 37% orthophosphoric acid (Scotchbond Universal Etchant, 3M Oral Care) for 15 s, rinsed with distilled water for 10 s, and air dried with a gentle oil-free air stream using the three-in-one syringe of a dental unit for 15 s. Scotch Bond Universal (3M Oral Care) was actively applied to the composite surfaces using a microbrush with a rubbing motion for 20 s. The sample was then air thinned with a gentle oil-free air stream using the three-in-one syringe of a dental unit for 5 s and subsequently light cured for 10 s with an LED light unit at 1000 mW/cm^2^.
Group D: Silane + Universal Adhesive (S+UA); a silane coupling agent (Ultradent Silane; South Jordan, UT, USA) was applied to roughened surfaces, allowed to react for 1 min, and then air dried with a gentle oil-free air stream using the three-in-one syringe of a dental unit for 30 s. Scotch Bond Universal (3M Oral Care) was applied as described above.
Group E: Cojet + Silane +Universal Adhesive (C+S+UA); composite surfaces were abraded with 30-µm silica-modified Al_2_O_3_ particles for 15 s with 2.5 bar pressure from a distance of approximately 10 mm. After Cojet application, silane and adhesive were applied as described above.

After surface treatment, a cylindrical mold (4 mm thick × 2 mm diameter) was placed on the treated composite surface and filled with uncured repair composite (Filtek Bulk-fill Flowable, 3M Oral Care). The repair composite was polymerized for 20 s with the LED curing light at 1000 mW/cm^2^. The repaired specimens were stored in distilled water at 37°C for 24 h and then subjected to the shear bond strength (SBS) test.

The shear bond strength test was performed in a universal testing machine (MIN 100, MOD Dental, Esetron Smart Robotechnologies; Ankara, Turkey) using a metal half-moon shear apparatus. The blade was positioned at an angle of 90 degrees to the repaired surface using a 1- kN cell at a 0.5 mm/min crosshead speed until failure. Shear bond strengths obtained in N were then calculated by dividing the maximum failure loads (in Newton) by the adhesive area of the specimens.

### Evaluation of Repair-Fresh Composite Interface using Scanning Electron Microscopy

For scanning electron microscopy analysis (SEM), one specimen from each group was randomly selected for SEM evaluation to observe the surface topography with a thickness of 4 mm and a diameter of 5 mm by applying the same surface treatments and repairs as in the study by Usumez et al.^51^ The repaired specimens were embedded to the autopolymerizing acrylic resin and then sectioned perpendicularly to the bonded interface using a low-speed diamond saw (Isomet, Buehler; Lake Bluff, IL, USA) to the center of the cylinder, under water cooling. All sectioned specimens were flattened using 400-, 800-, 1200-, 2400-, and 4000-grit SiC-abrasive papers, followed by polishing with diamond particles of 3.1 and 0.25 μm. After the flattening procedure, the specimens were cleaned by ultrasonication after each polishing step to remove the debris and sputter-coated with gold using a vacuum coating machine (Leica EM ACE200, Leica Microsystems; Wetzlar, Germany), then examined using environmental scanning electron microscopy ([E-SEM] Hitachi SU5000 FE-SEM; Tokyo, Japan) employing the back-scatter technique. The E-SEM analysis was performed at 10 kV, with water vapor as the imaging gas in the sample chamber, held at a pressure of 1.0 Torr and 5000X magnification.

Statistical analysis was performed using SPSS version 22 software (IBM; Armonk, NY, USA). Data were analyzed for normality using the Kolmogorov-Smirnov test and the Shapiro-Wilk test. It was determined that the parameters were normally distributed. The effect of the interaction of composite and surface treatment on shear bond strength was evaluated using two-way ANOVA, and Tukey’s HSD test was used in post-hoc analyses. Statistical significance was set at p<0.05.

### Data Availability

The datasets generated and analyzed in the current study are available from the corresponding author upon reasonable request.

## RESULTS 

Two-way ANOVA revealed statistically significant effects of both surface treatment and composite type on shear bond strength (SBS) (p < 0.05). The mean SBS values and statistical comparisons for all experimental groups are presented in Table 2.

**Table 2:** Mean and standard deviations (SD) SBS test values of the groups

	Filtek Bulk-Fill Flowable	Surefil SDR	EverX Flow	p
	Mean ±SD	Mean±SD	Mean ±SD	
Group A: control	10.42±0.47^aA^	11.42±1.03^aA^	7.36±1.73^bA^	0.001*
Group B: UA	12.31±1.35^aB^	13.95±1.58^bB^	12.85±0.65^abB^	0.022*
Group C: A + UA	13.65±1.50^aB^	13.70±1.31^aB^	13.57±1.26^aB^	0.977
Group D: S+UA	15.49±1.12^aC^	16.84±1.22^bC^	15.80±0.96^abC^	0.029*
Group E: C+S+UA	19.59±1.30^aD^	20.27±1.44^aD^	18.98±1.01^aD^	0.092
p	0.001*	0.001*	0.001*	
*Two-way ANOVA p<0.05. Different lowercase letters in the rows indicate differences between composite groups. Different uppercase letters in the columns indicate differences between surface treatment groups.				

### Effect of Surface Treatment on Shear Bond Strength

Surface treatment statistically significantly affected SBS within each of the three bulk-fill resin composites (p = 0.001). For all three materials, the untreated control group (Group A) exhibited the lowest SBS, whereas CoJet + silane + universal adhesive (Group E) produced the highest values (p < 0.05).

Application of universal adhesive alone (Group B) statistically significantly increased SBS compared with the untreated control (Group A) for all three composites (p < 0.05). The addition of phosphoric acid etching before universal adhesive application (Group C) did not result in a statistically significant difference compared with universal adhesive alone (Group B) (p > 0.05). In contrast, the application of silane before the universal adhesive (Group D) resulted in statistically significantly higher SBS than in Groups B and C (p < 0.05). CoJet treatment followed by silane and universal adhesive (Group E) produced statistically significantly higher SBS than all other surface treatment protocols for each composite (p < 0.05).

### Effect of Composite Type within Each Surface Treatment

The effect of composite type varied according to the surface treatment protocol. In the untreated control group (Group A), EverX Flow exhibited statistically significantly lower SBS than did Filtek Bulk-Fill Flowable and Surefil SDR (p = 0.001), whereas no statistically significant difference was observed between Filtek Bulk-Fill Flowable and Surefil SDR.

In the universal adhesive group (Group B), a statistically significant difference was detected among the three composites (p = 0.022). Surefil SDR exhibited significantly higher SBS than did Filtek Bulk-Fill Flowable, while EverX Flow showed an intermediate value that did not differ statistically significantly from either material.

Similarly, in the silane + universal adhesive group (Group D), a statistically significant difference was observed among the composites (p = 0.029). Surefil SDR exhibited statistically significantly higher SBS than Filtek Bulk-Fill Flowable, whereas EverX Flow showed an intermediate value without a statistically significant difference from either material.

No statistically significant differences among Filtek Bulk-Fill Flowable, Surefil SDR, and EverX Flow were observed following phosphoric acid + universal adhesive treatment (Group C; p = 0.977) or CoJet + silane + universal adhesive treatment (Group E; p = 0.092).

## DISCUSSION

The aim of repair procedures in restorative dentistry is to establish a strong and durable bond between the existing restoration and new composite material while ensuring a minimally invasive approach. Numerous materials, techniques, and surface treatments have been studied to enhance the bond strength of repaired composite surfaces, including chemical and physical methods.^10,27,35^ However, there is no consensus on the optimal surface treatment protocol for aged restorations.

One major challenge in composite repair is the absence of an oxygen-inhibited layer and the depletion of unreacted methacrylate double bonds (C=C) on aged composite surfaces, which are essential for chemical bonding with new composite layers.^26^ Composite restorations age naturally in the oral environment, exposed to temperature fluctuations, enzymes, bacteria, and fluids, all contributing to degradation.^49^ Although in-vitro aging methods vary, thermocycling is widely accepted, with 10,000 cycles simulating approximately one year of oral aging.^21^ This study used thermocycling to simulate aging before repair procedures. Flowable bulk-fill resin composites are favored for their ease of application, especially in cavities with limited access, due to their syringe delivery and good adaptation to cavity walls.^46^ These composites generally have lower filler content and reduced wear resistance, thus often requiring a capping layer made from conventional resin or high-viscosity bulk-fill resin composites.^13^ However, some flowable bulk-fills, like 3M Filtek Bulk-Fill Flowable used in this study, can function as full-body restorative materials.

This study evaluated the effects of different surface treatment protocols, universal adhesives, and silane applications on the repair bond strength of aged bulk-fill resin composites. Our results demonstrated that all tested repair protocols statistically significantly increased shear bond strength compared to untreated controls, leading to rejection of the null hypothesis that surface treatment does not affect bond strength. Acid etching remains a cornerstone in restorative dentistry for improving adhesion by increasing micromechanical retention.^36^ Both phosphoric acid and hydrofluoric acid have been extensively used to enhance surface roughness, improving bond strength and restoration survival.^20^ However, due to hydrofluoric acid’s corrosive nature and associated safety risks, its clinical use has diminished, with phosphoric acid or air-abrasion techniques such as CoJet being preferred alternatives.^41^ Phosphoric acid selectively dissolves enamel and dentin minerals, increasing surface energy and creating microretentive structures that promote hybrid layer formation, especially in enamel.^19^ In this study, 37% phosphoric acid applied for 15 s statistically significantly improved bond strength compared to controls, likely by effectively removing debris and contaminants.^35^ However, since repair surfaces lacked enamel or dentin substrates, no statistically significant difference was observed between universal adhesive application with or without prior acid etching.

Universal adhesives contain both hydrophilic and hydrophobic monomers, allowing versatile use in self-etch, selective-etch, or total-etch modes.^23^ Their key component, 10-methacryloyloxydecyl dihydrogen phosphate (10-MDP), chemically bonds to calcium in hydroxyapatite and metal oxides, enhancing durability.^54^ Many universal adhesives also include silane coupling agents, facilitating adhesion to silica-based ceramics and composite fillers without a separate silanization step, streamlining clinical procedures.^56^

Among the bulk-fill resin composites tested, Surefil SDR showed comparatively high repair bond strength. This may be attributed to its modified urethane dimethacrylate (UDMA) resin matrix, which relieves polymerization stress and provides a stable substrate.^29^ Its lower filler content and increased translucency may also contribute to differences in its interfacial behavior.^34^ The comparatively higher repair bond strength observed for SDR in the present study is consistent with previously reported material-dependent differences in the bonding performance of bulk-fill resin composites.^17^

EverX Flow generally exhibited lower repair bond strength values than the other composites, with a statistically significant difference observed in the untreated control group. Although polymerization shrinkage or stress formation was not evaluated in the present study, earlier research has shown that the resin matrix composition and fiber distribution of SFRCs can influence interfacial behavior and bonding performance. Lassila et al^33^ reported that SFRCs exhibit material-dependent differences in interfacial stress development and bonding interactions due to their modified viscosity and fiber architecture. Garoushi et al^22^ further demonstrated that the fiber-reinforced structure contributes to improved fracture toughness but may also affect adaptation at the bonding interface depending on fiber orientation and matrix composition. Therefore, the lower repair bond strength observed for EverX Flow in this study may be related to its material-specific structural and compositional characteristics, rather than any directly measured parameter. The universal adhesive used contains 10-MDP and silane coupling agents, enhancing chemical bonding to silica-containing fillers, which may explain better bonding in silica-rich materials like SDR and Filtek Bulk-Fill Flowable.^8^ However, as noted by Lasilla et al,^32^ modifications in viscosity and composition also results in increased polymerization shrinkage, which is associated with conventional flowable composites. Induced shrinkage in resin composites may generate higher interfacial stress during polymerization, potentially compromising the bond strength of the repair. Furthermore, considering the statistically significant differences observed among the bulk-fill resin composites tested, the second null hypothesis, which stated that the type of bulk-fill resin composite does not influence the repair efficacy, was also rejected.

This study also found that silane applications statistically significantly improved repair bond strength. Silanes are bifunctional molecules that chemically link organic resin and inorganic filler phases, strengthening the repair interface.^4^ The most commonly used dental silane is 3-methacryloxypropyl-trimethoxysilane (MPS), which bonds to resin composites and forms siloxane bonds with silica or alumina on pretreated surfaces.^50^ Silanes exist in hydrolyzed and non-hydrolyzed forms; the former can be applied separately or included in universal adhesives.^39^ Re-silanization is especially important in aged composites where the original silane layer has degraded, as it improves adhesive diffusion and durability.^28^ These agents are bifunctional molecules that possess a nonhydrolyzable functional group capable of co-polymerizing with the methacrylate groups in the organic matrix of resin composite and hydrolyzable alkoxy groups that react with hydroxyl groups on the surface of silica-based fillers to form stable oxygen bridges.^4^ This dual functionality allows silanes to chemically link the organic and inorganic phases, thereby strengthening the interface between the aged composite substrate and the new repair material.^28^ Several studies have demonstrated that application of silane increases surface wettability, which not only promotes better adhesive penetration into surface defects and micro-retentions but also enhances the chemical bonding to exposed filler particles.^37,42,53^ In aged composites, where degradation and loss of the original silane layer is expected, this re-silanization process is particularly effective.^28^ The re-silanization method ensures improved diffusion of the bonding agent, significantly contributing to the durability and strength of the repaired interface.^47^

The application of the Cojet system to repair the surface yielded the highest SBS results among all repair techniques in our studies. Similar to our results, all studies agree that mechanical surface treatment with air abrasion shows promising results.^37,45^

As observed in the representative SEM images of the composite–composite interface, CoJet surface conditioning produced a more irregular and microretentive surface morphology compared with the other surface conditioning protocols (Fig 1). In the representative SEM image of the CoJet-treated EverX specimen, alterations in the morphology of the exposed glass-fiber-containing structure were observed. These morphological features may reflect the interaction between airborne-particle abrasion and the heterogeneous fiber-reinforced surface of EverX Flow. However, as the present SEM evaluation was qualitative, further studies specifically designed to characterize the effect of airborne-particle abrasion on glass-fiber integrity are warranted.

**Fig 1:**
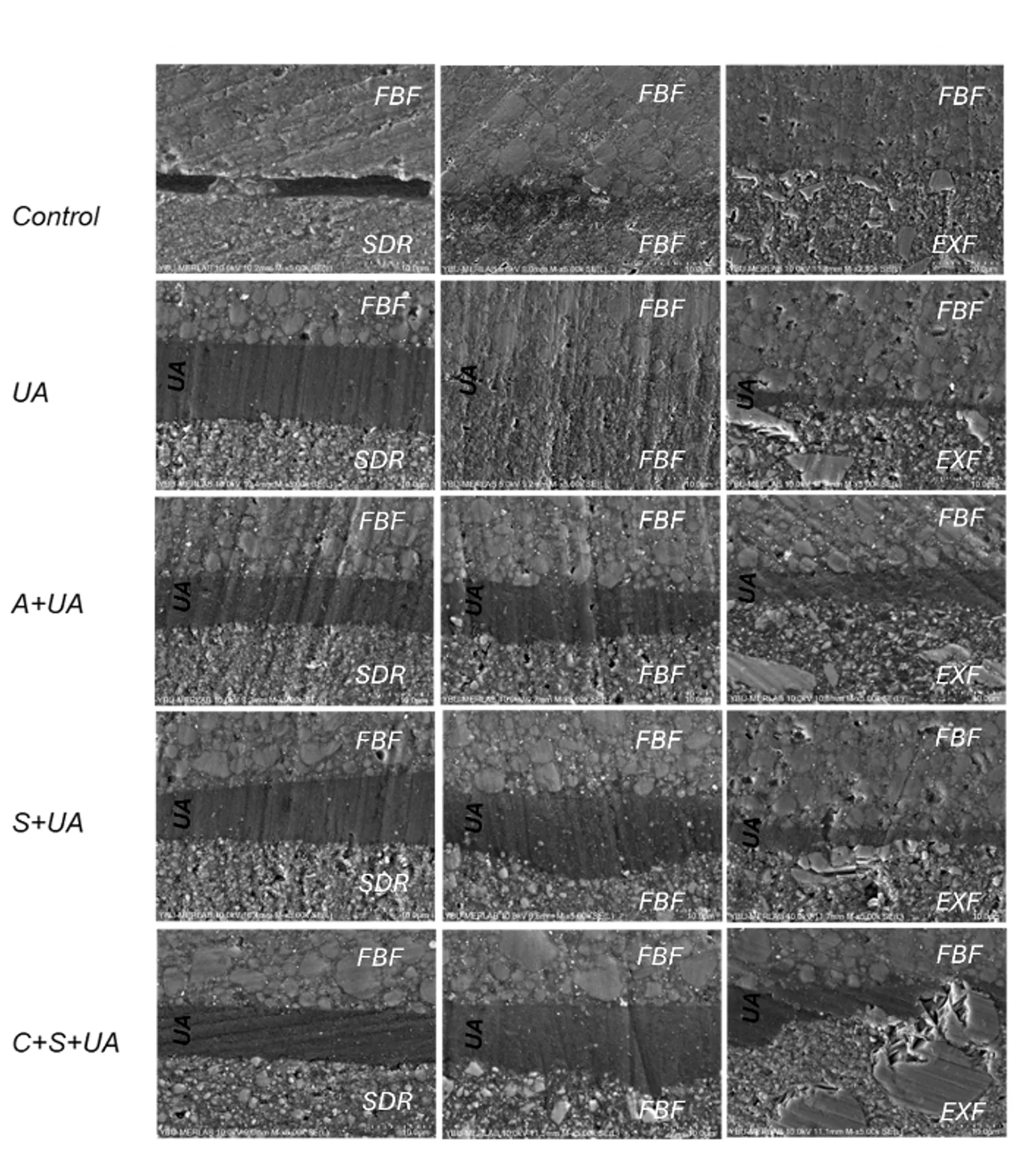
SEM images show selected samples of surface treatment groups of bulk-fill restorative materials. FBF: Filtek Bulk-fill Flowable. SDR: Surefil SDR. EXF: EverX Flowable. UA: Universal Adhesive.

Therefore, although rubber-dam is used as a dust-catcher, the safety of this material is controversial because the aerosol formed by the fine abrasive particles in its structure can be harmful to the operator and the patient.^24^ The high SBS observed following CoJet + silane + universal adhesive treatment may be associated with the combined micromechanical effect of airborne-particle abrasion and chemical interaction between the additional silane and exposed silica-containing surfaces.

This in-vitro study does not fully replicate the complexity of the oral environment, including variations in saliva composition, pH, biofilm, and functional loading. Thermocycling was performed with distilled water instead of artificial saliva, which may influence aging outcomes. Additionally, polymerization shrinkage, interfacial stress development, and long-term bond durability were not evaluated. Future research should incorporate more clinically relevant aging protocols, mechanical loading, and extended storage periods, as well as in-vivo studies to validate the present findings.

## CONCLUSION

All surface treatment protocols improved the repair bond strength of aged bulk-fill resin composites compared to untreated controls. Silane application and the CoJet system produced the highest values, indicating their effectiveness in enhancing micromechanical and chemical adhesion. Although some material-dependent differences were observed in certain treatment groups, no statistically significant difference was detected among the three bulk-fill resin composites when CoJet, silane, and universal adhesive were combined (Group E, p = 0.092). This suggests that when an optimized surface conditioning protocol is used, substrate composition may play a less dominant role in repair performance. Further studies under more clinically relevant conditions are needed to confirm the durability and clinical applicability of these findings.

The highest repair bond strength for aged bulk-fill composites is achieved by combining CoJet air abrasion, silane, and universal adhesive to maximize micromechanical and chemical interlocking. While phosphoric acid etching alone is insufficient, a separate silanization step significantly improves surface wettability and chemical bonding to exposed fillers. This optimized protocol ensures reliable repair outcomes across various bulk-fill materials, effectively extending restoration longevity through minimally invasive procedures.

### AI Statement

No artificial intelligence (AI) tools were used at any stage of the research project, data analysis, preparation, or writing of the manuscript. Therefore, no AI-assisted content is included in the manuscript.
